# Draft Genome Sequence of a Salmonella enterica subsp. *enterica* Serotype Choleraesuis Strain Isolated from the Pulp of Muskmelons

**DOI:** 10.1128/MRA.00009-21

**Published:** 2021-03-11

**Authors:** Irene Esteban-Cuesta, Jennie Fischer, Claudia Guldimann

**Affiliations:** a Chair of Food Safety and Analytics, Ludwig-Maximilians University Munich, Oberschleissheim, Germany; b German Federal Institute for Risk Assessment (Bundesinstitut für Risikobewertung, BfR), National Salmonella Reference Laboratory, Food Microbiology, Host-Pathogen-Interactions Unit, Department of Biological Safety, Berlin, Germany; University of Maryland School of Medicine

## Abstract

Salmonella enterica subsp. *enterica* serotype Choleraesuis is a foodborne pathogen with zoonotic potential. We report the draft genome sequence and a closed plasmid sequence from a plant-internalized S. Choleraesuis strain that was isolated from the pulp of a Spanish Galia melon purchased from a German supermarket in 2015.

## ANNOUNCEMENT

Salmonella enterica subsp. *enterica* serotype Choleraesuis bacteria are host adapted to pigs. However, human infections do occur and typically result in invasive disease (1). Transmission to humans might result from fecal contamination of plants that are grown close to the ground and consumed raw, such as salad, spinach, or melons (2–4). Several foodborne outbreaks caused by melons contaminated with *Salmonella* spp. have been reported (5, 6), and internalization into plants has been described for *Salmonella* spp. (7–9). This may offer the pathogen a growth advantage and therefore increase the risk to consumers. Strain 11-Galia-2015 was isolated from the pulp of a Galia melon (8) and serotyped at the German Federal Institute for Risk Assessment (BfR). Here, we present the draft whole-genome sequence of this strain.

Genomic DNA was extracted from a liquid culture grown in LB medium using the PureLink genomic DNA minikit (Themo Fisher Scientific, Germany). A sequencing library was created with the Nextera DNA Flex library prep kit (Illumina, USA) and sequenced using 2 × 201-bp format on a MiSeq benchtop sequencer with v3 600-cycle chemistry (Illumina), resulting in 1,585,934 total reads and 630-fold coverage. The generated paired-end reads (Q30 base fraction, 0.95) were trimmed and quality controlled using fastp v0.19.5 (10) with default parameters (except --length_required 15) and *de novo* assembled using Shovill v1.1.0 (10, 11) using SPAdes as the assembly method (with the options --noreadcorr --depth 100 and otherwise default parameters). The minimal contig size was set to 200 bp. Annotation of S. Choleraesuis with the antigenic profile 7:c:1,5 (SeqSero v1.2 Server [11]) was performed using PGAP (12). However, serological subtyping according to the White-Kauffmann-Le Minor scheme (13) resulted in the antigenic profile 6,7:−:1,5.

The assembled S. Choleraesuis genome sequence comprised 79 contigs with a total length of 4,723,419 bp, an *N*_50_ value of 216,747 bp, and 52.2% GC content. In total, 4,386 coding genes were predicted in the chromosome, including 23 rRNAs (5S, 16S, and 23S), 79 tRNAs, and 10 noncoding RNAs (ncRNAs).

*In silico* Multi-Locus Sequence Typing (MLST) v2.0 (14) (https://cge.cbs.dtu.dk/services/MLST/) identified this strain as sequence type 145, and cgMLSTFinder v1.1 (15) (https://cge.cbs.dtu.dk/services/cgMLSTFinder/) identified it as core genome sequence type (cgST) 168374.

PlasmidFinder (16) identified one complete circular multireplicon IncFIB(S)-FII(S) plasmid (p11-Galia-2015), with 49,465 bp and 52.2% GC content. ResFinder v4.1 (17, 18) found the aminoglycoside resistance gene *aac(6′)-laa* against amynoglycosides, not located on the plasmid.

A total of 111 virulence factors were identified using ABRicate v1.0.1 (https://github.com/tseemann/abricate) (with the parameters --minid 80 and --mincov 50). Among them, major virulence determinants belonging to *Salmonella* pathogenicity island 1 (SPI-1; e.g., *inv*, *sip*, *sptP*, *sopA*, and *sic*), SPI-2 (e.g., *ssa*, *ssc*, *sse*, *spiC*, *sifA*, *pipB*, and *sopD2*), and SPI-3 (*mgtCB* and *misL*) were found. Also, genes coding for virulence factors, such as *sodCI*, which protects against phagocytic superoxide during infection (19), Peyer’s patch-specific virulence factors (*lpfA-E*), and the toxin-coding genes *spvB* or *mig-14*, responsible for resistance to antimicrobial peptides, were identified (20, 21).

### Data availability.

These sequences are available at GenBank (accession number JADWDF000000000 for the genome) and the SRA (accession number SRR13399484). The version described in this paper is the first version, JADWDF010000000.

## References

[1] Chiu C-H, Su L-H, Chu C. 2004. Salmonella enterica serotype Choleraesuis: epidemiology, pathogenesis, clinical disease, and treatment. Clin Microbiol Rev 17:311–322. doi:10.1128/cmr.17.2.311-322.2004. 15084503 PMC387403

[2] Centers for Disease Control and Prevention. 2020. List of selected multistate foodborne outbreak investigations. Centers for Disease Control and Prevention, Atlanta, GA. https://www.cdc.gov/foodsafety/outbreaks/multistate-outbreaks/outbreaks-list.html.

[3] EFSA, European Food Safety Authority, Panel on Biological Hazards (BIOHAZ). 2014. Scientific opinion on the risk posed by pathogens in food of non-animal origin. Part 2 (Salmonella in melons). EFSA J 12:3831. doi:10.2903/j.efsa.2014.3831. PMC13136943 42087975

[4] EFSA, European Food Safety Authority. Panel on Biological Hazards (BIOHAZ). 2013. Scientific opinion on the risk posed by pathogens in food of non-animal origin. Part 1 (outbreak data analysis and risk ranking of food/pathogen combinations). EFSA J 11:3025. doi:10.2903/j.efsa.2013.3025. PMC13159936 42124987

[5] Byrne L, Fisher I, Peters T, Mather A, Thomson N, Rosner B, Bernard H, McKeown P, Cormican M, Cowden J, Aiyedun V, Lane C, International Outbreak Control Team. 2014. A multi-country outbreak of Salmonella Newport gastroenteritis in Europe associated with watermelon from Brazil, confirmed by whole genome sequencing: October 2011 to January 2012. Euro Surveill 19:6–13. doi:10.2807/1560-7917.es2014.19.31.20866. 25138971 PMC4516299

[6] CDC. 2002. Multistate outbreaks of Salmonella serotype Poona infections associated with eating cantaloupe from Mexico—United States and Canada, 2000–2002. MMWR Morb Mortal Wkly Rep 51:1044–1047. 12487526

[7] Klerks MM, van Gent-Pelzer M, Franz E, Zijlstra C, van Bruggen AHC. 2007. Physiological and molecular responses of Lactuca sativa to colonization by Salmonella enterica serovar Dublin. Appl Environ Microbiol 73:4905–4914. doi:10.1128/AEM.02522-06. 17513585 PMC1951040

[8] Esteban-Cuesta I, Drees N, Ulrich S, Stauch P, Sperner B, Schwaiger K, Gareis M, Gottschalk C. 2018. Endogenous microbial contamination of melons (Cucumis melo) from international trade: an underestimated risk for the consumer? J Sci Food Agric 98:5074–5081. doi:10.1002/jsfa.9045. 29604072

[9] Gu G, Hu J, Cevallos-Cevallos JM, Richardson SM, Bartz JA, van Bruggen AHC. 2011. Internal colonization of Salmonella enterica serovar Typhimurium in tomato plants. PLoS One 6:e27340. doi:10.1371/journal.pone.0027340. 22096553 PMC3212569

[10] Chen S, Zhou Y, Chen Y, Gu J. 2018. fastp: an ultra-fast all-in-one FASTQ preprocessor. Bioinformatics 34:i884–i890. doi:10.1093/bioinformatics/bty560. 30423086 PMC6129281

[11] Zhang S, Yin Y, Jones MB, Zhang Z, Deatherage Kaiser BL, Dinsmore BA, Fitzgerald C, Fields PI, Deng X. 2015. Salmonella serotype determination utilizing high-throughput genome sequencing data. J Clin Microbiol 53:1685–1692. doi:10.1128/JCM.00323-15. 25762776 PMC4400759

[12] Zhao Y, Wu J, Yang J, Sun S, Xiao J, Yu J. 2012. PGAP: pan-genomes analysis pipeline. Bioinformatics 28:416–418. doi:10.1093/bioinformatics/btr655. 22130594 PMC3268234

[13] Grimont PAD, Weill F-X. 2007. Antigenic formulae of the Salmonella serovars, 9th ed. Institute Pasteur, Paris, France.

[14] Larsen MV, Cosentino S, Rasmussen S, Friis C, Hasman H, Marvig RL, Jelsbak L, Sicheritz-Pontén T, Ussery DW, Aarestrup FM, Lund O. 2012. Multilocus sequence typing of total-genome-sequenced bacteria. J Clin Microbiol 50:1355–1361. doi:10.1128/JCM.06094-11. 22238442 PMC3318499

[15] Clausen PTLC, Aarestrup FM, Lund O. 2018. Rapid and precise alignment of raw reads against redundant databases with KMA. BMC Bioinformatics 19:307. doi:10.1186/s12859-018-2336-6. 30157759 PMC6116485

[16] Carattoli A, Zankari E, García-Fernández A, Voldby Larsen M, Lund O, Villa L, Aarestrup FM, Hasman H. 2014. In silico detection and typing of plasmids using PlasmidFinder and plasmid multilocus sequence typing. Antimicrob Agents Chemother 58:3895–3903. doi:10.1128/AAC.02412-14. 24777092 PMC4068535

[17] Bortolaia V, Kaas RS, Ruppe E, Roberts MC, Schwarz S, Cattoir V, Philippon A, Allesoe RL, Rebelo AR, Florensa AF, Fagelhauer L, Chakraborty T, Neumann B, Werner G, Bender JK, Stingl K, Nguyen M, Coppens J, Xavier BB, Malhotra-Kumar S, Westh H, Pinholt M, Anjum MF, Duggett NA, Kempf I, Nykäsenoja S, Olkkola S, Wieczorek K, Amaro A, Clemente L, Mossong J, Losch S, Ragimbeau C, Lund O, Aarestrup FM. 2020. ResFinder 4.0 for predictions of phenotypes from genotypes. J Antimicrob Chemother 75:3491–3500. doi:10.1093/jac/dkaa345. 32780112 PMC7662176

[18] Zankari E, Allesøe R, Joensen KG, Cavaco LM, Lund O, Aarestrup FM. 2017. PointFinder: a novel Web tool for WGS-based detection of antimicrobial resistance associated with chromosomal point mutations in bacterial pathogens. J Antimicrob Chemother 72:2764–2768. doi:10.1093/jac/dkx217. 29091202 PMC5890747

[19] De Groote MA, Ochsner UA, Shiloh MU, Nathan C, McCord JM, Dinauer MC, Libby SJ, Vazquez-Torres A, Xu Y, Fang FC. 1997. Periplasmic superoxide dismutase protects Salmonella from products of phagocyte NADPH-oxidase and nitric oxide synthase. Proc Natl Acad Sci U S A 94:13997–14001. doi:10.1073/pnas.94.25.13997. 9391141 PMC28421

[20] Chen L, Yang J, Yu J, Yao Z, Sun L, Shen Y, Jin Q. 2005. VFDB: a reference database for bacterial virulence factors. Nucleic Acids Res 33:D325–D328. doi:10.1093/nar/gki008. 15608208 PMC539962

[21] Singh Y, Saxena A, Kumar R, Kumar Saxena M. 2018. Virulence system of Salmonella with special reference to Salmonella enterica. *In* Mascellino MT (ed), Salmonella—a re-emerging pathogen. IntechOpen, London, United Kingdom.

